# No Biological Evidence of XMRV Infection in Cervical Smears from HIV/ HPV Positive and Negative Kenyan Women

**DOI:** 10.1371/journal.pone.0047208

**Published:** 2012-10-08

**Authors:** Xiaotong He, Thomas D. J. Walker, Innocent O. Maranga, Anthony W. Oliver, Lynne Hampson, Ian N. Hampson

**Affiliations:** 1 Viral Oncology Laboratories, University of Manchester School of Cancer & Enabling Sciences, St Mary's Hospital, Manchester, United Kingdom; 2 Departments of Obstetrics and Gynaecology, University of Nairobi, Nairobi, Kenya; Baylor College of Medicine, United States of America

## Abstract

**Background:**

XMRV (xenotropic murine leukaemia virus-related virus) is a gammaretrovirus first discovered in human prostate carcinomas and later linked to chronic fatigue syndrome (CFS). Emerging conflicting data and lack of reproducibility of results within the scientific community has now led to the association of XMRV with CFS being discounted. Indeed the case for an involvement with any human disease has been questioned with the suggestion that XMRV is a laboratory generated recombinant virus. The fact that not all published positive findings can be easily explained as contamination artefacts coupled with the observation that XMRV may have a sexually transmitted mode of infectivity and can be infectious for primates, where it preferential resides in cells of the reproductive tract, prompted us to look for evidence of XMRV in the cervical cells of a cohort of Kenyan women both with and without pre-existing HIV/HPV infections.

**Results:**

Using a highly sensitive and selective triplex PCR approach we analysed DNA from the liquid based cytology (LBC) cervical smears of 224 Kenyan women. There was no evidence of XMRV expression in any of the sample population irrespective of HPV and/or HIV status.

**Conclusions:**

The data presented show no indication of XMRV infection in any of the cervical samples screened in this study. Approximately 50% of the women were HIV positive but this did not influence the findings signifying that XMRV does not act as an opportunistic infection in this cohort nor is it related to HPV status. Our results therefore support the findings that XMRV is confined to the laboratory and does not currently represent an infectious agent for humans, with a cautionary adage that such potential zoonotic viruses should be carefully monitored in the future.

## Introduction

In 2006 a new gammaretrovirus Xenotropic murine leukaemia virus-related retrovirus (XMRV) was discovered in human prostate carcinoma tissues, representing the first potentially pathogenic gammaretroviral infection of humans [1]. Initially the virus was isolated from the prostatic tumours of patients who had missense mutations in the gene encoding RNASEL (mutation designated R462Q), a protein involved in several functions including the cellular anti-viral response. The R462Q mutation was known to have reduced RNASEL enzymatic activity and had already been previously shown to be associated with prostate cancer [2]. The discovery of XMRV in RNASEL mutant tumours demonstrated an association between XMRV infection and RNASEL deficiency but did not suggest any direct link of the virus to prostate cancer at this stage. This was reported later by others though, who also correlated levels of XMRV to disease severity [3]. Work rapidly progressed in the area and soon XMRV was also being linked to chronic fatigue syndrome (CFS) [4].

However, failure by many laboratories to reproduce the XMRV and CFS findings seriously undermined the existence of a link between the two with the resulting outcome being retraction of the original finding paper [5]. Indeed the case for XMRV being associated with any type of disease started to be questioned, with the suggestion by many that positive XMRV results achieved by PCR based methods were due to sample contamination with mouse DNA or other sources such as infected cell-line DNA or viral particles [6]. However not all of the studies showing positivity of XMRV in prostate tissue relied on PCR methodologies and instead adopted a serological approach [1], [3]. The controversy continues, but one thing which is clear is that the virus does exist and as such is a putative zoonotic capable of infecting human tissues. Indeed XMRV has been shown in cell culture to have a broad human tissue tropism including cervical cells where it was found to be able to replicate efficiently [7]. Furthermore, recently published work by Sharma *et al* indicates that XMRV is infectious for primates and preferentially concentrates in cells of the reproductive tract suggesting sexual transmission as a potential route of infectivity. XMRV positivity, by immunohistochemistry, in the female simian is focused in the cells of the cervical epithelium and submucosal cells of the vagina [8].

This predilection of the virus with cells of the reproductive tract prompted us to look at whether it could be detected in human cervical tissue and what influence, if any, the presence of the Human Papilloma Virus (HPV) and/or Human Immunodeficiency Virus (HIV) had on XMRV expression. No such study has yet been reported in the literature. As has been suggested for XMRV both HPV and HIV are sexually transmitted and infection with either virus is known to facilitate infection with the other [9], [10], [11]. Indeed, HIV infection has been associated with the activation of many opportunistic pathogenic infections, thought mainly to be due to dysfunctional immune responses, and molecular interactions between HIV and these pathogens are likely to have a role in disease progression [12]. Thus any potential relationship between XMRV positivity and HIV and/or HPV status would be a significant finding. Furthermore, the reported link between XMRV expression and RNASEL variant R462Q suggests that XMRV expression should be looked for in cervical smears, as germline mutations in RNASEL have also been shown to predict an increased risk of cervical cancer [13].

Here we analyse by PCR the expression of XMRV in DNA extracted from LBC cervical samples from a cohort of 224 Kenyan women. Half these women are also HIV positive and approximately 50% of them are infected with HPV. We find no evidence for XMRV in any of the patient samples.

## Materials and Methods

### Collection of LBC cervical samples

224 samples were collected with written informed consent from 113 HIV+ve and 111 HIV-ve Kenyan women who attended the Specialist HIV Clinic and Family Planning Clinic at Kenyatta National Hospital between April 2008 and February 2009. The women ranged in age between 21 and 52 years (median age: 35 years) and those who had prior destructive procedures for cervical disease and hysterectomies were excluded. Cervical samples were collected into PreservCyt transport solution (ThinPrep Pap Test, Hologic Inc, USA).

### Ethics Statement

Ethical approval was granted by both the Kenyatta National Hospital Ethics Committee (05.12.2007: KNH-ERC/01/4988) and the Oldham Ethics Committee UK (26.01.2009: amendment 5 project 07/Q1405/14).

### DNA Extraction

Automated DNA extraction was performed on 500 µl of all 224 PreserveCyt LBC samples with the use of a BioRobot® M48, (Qiagen, Sussex, UK) by an accredited team within the Manchester Virology Laboratory, St Mary's Hospital, Manchester, UK. The integrity of each DNA sample was validated by two internal controls during subsequent analysis with the Papillocheck system (Greiner Bio-One Ltd, Stonehouse, UK) - manuscript in preparation.

### Genomic DNA Integrity Validation

Prior to PCR analysis DNA samples were further validated by detection of the housekeeping gene glyceraldehyde 3-phosphate dehydrogenase *(GAPDH)* (NCBI accession number: NM_002046). The primers (forward primer sequence: HM-GAPDH-F CATTGACCTCAACTACATGGT; reverse primer sequence: HM-GAPDH-R TCGCTCCTGGAAGATGGTGAT) generate amplicons of 130 bp.

Standard 50 µl reactions were prepared which comprised 2 µl of 25 ng/µl template DNA, 2.5 units of BioTaq DNA polymerase (Bioline Ltd, London, UK), 0.2 mM dNTPs, and 0.2 µM of each primer in 10 mM Tris-HCL pH 8.3, 50 mM KCl, and 2.5 mM MgCl_2_. Thermocycler conditions comprised an activation stage of 94°C for 5 minutes; 33 cycles of 94°C×25 seconds, 53°C×25 seconds, 72°C×25 seconds; and a final extension of 72°C for 7 minutes. All reactions were performed on a Verity® 96-well Fast Thermo Cycler (Applied Biosystems, Paisley, UK).

20 µl of PCR product was loaded into 2% w/v agarose gels (SeaKem® LE Agarose, Lonza, Rockland, USA) and separated by agarose gel electrophoresis (Clarit-E Maxi electrophoresis tanks, Alpha Laboratories, Hampshire, UK) at 60 volts for 90 minutes. DNA was visualised using ethidium bromide and imaged on a UVP Transilluminator (California, USA).

### Single-run Triplex PCR

A single-run, hot-start, touch-down triplex PCR assay was optimized with the use of a multiplex PCR kit (Qiagen, Sussex, UK) and three primer sets specific to XMRV (XMRV NCBI accession number: DQ399707). The first primer set generates amplicons of 458 bp from within the *gag* region of the XMRV sequence;

(forward primer: XMRV-multiplex gag-F TCTGAGTCTAACCTTGCAGC; reverse primer: XMRV-multiplex gag-R GATCCTCTGTGAGAAGGTCA). The second primer set generates amplicons of 510 bp from within the *pol* region of the XMRV sequence

(forward primer: XMRV-multiplex pol-F CGAGCCGAACTGATAGCACTCA; reverse primer: XMRV-multiplex pol-R TCTGTGTAGGGAGTCTAACAGT).

The third primer set generates amplicons of 782bp from within the *env* region of the XMRV sequence (forward primer: XMRV-multiplex env-F TGACAGGACAAACAGCTAAT; reverse primer XMRV-multiplex env-R GCCAGCACTCTTGGGTTTTGTC).

Standard 25 µl reactions were prepared under conditions recommended by the manufacturer. Thermocycling conditions comprised an activation stage of 95°C for 15 minutes; 8 cycles of 94°C×1 minute, 62°C −1/cycle ×1.5 minutes, 72°C×1 minute; 37 cycles of 94°C×1 minute , 58°C×1 minute, 72°C×1 minute; and a final extension of 53°C for 7 minutes.

All reactions were prepared in duplicate or triplicate, and were performed on a Verity® 96-well Fast Thermo Cycler (Applied Biosystems, Paisley, UK). 20 µl of PCR product was loaded into 1.5% w/v agarose gels (SeaKem® LE Agarose, Lonza, Rockland, USA) and separated by 1% agarose gel electrophoresis (Clarit-E Maxi electrophoresis tanks, Alpha Laboratories, Hampshire, UK) at 60 volts for 90 minutes. DNA was visualised using ethidium bromide and imaged on a UVP Transilluminator (California, USA).

## Results

### Validation of Multiplex PCR Method

**Figure 1:**
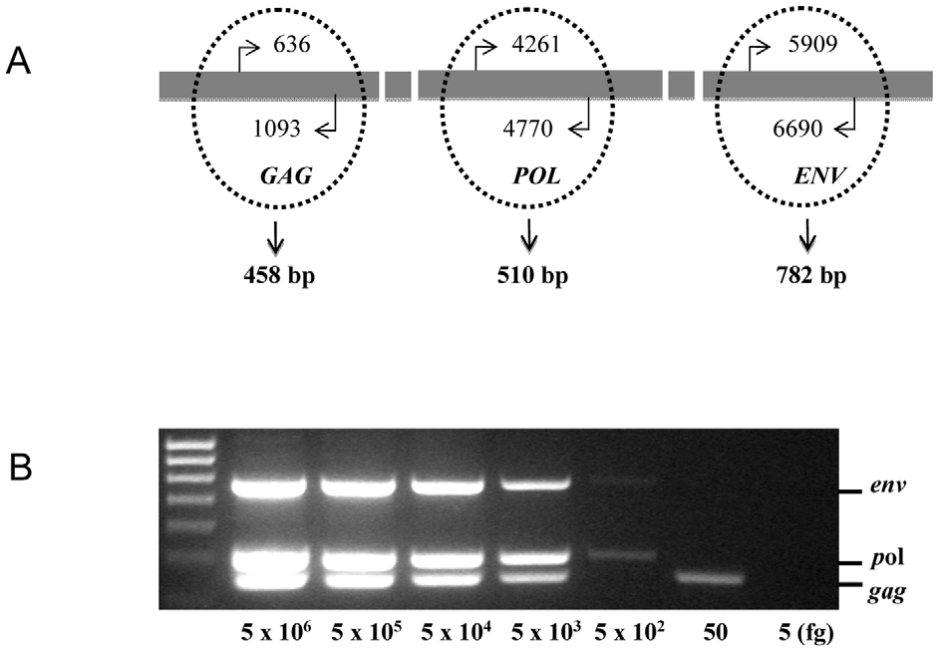
Establishment of a triplex PCR method for XMRV detection. (A) Schematic to show the areas of XMRV selected for priming triplex PCR. XMRV amplified sequences were targeted to the *gag, pol* and *env* genes, resulting in three potential amplicons of 458-bp, 510-bp and 782-bp respectively. (B) Assessment of the limits of XMRV detection by triplex PCR. A 5 ng −5 fg 22Rv1 DNA standard dilution was used to detect amplimers within the *env, pol,* and *gag* genes of XMRV by hot-start touch-down 45 cycle multiplex PCR. 5 pg of input template was sufficient for triplex signals; 0.5 pg for duplex signals, 0.05 pg for a singleplex signal.

Mutations in any particular target XMRV sequence could potentially produce false negative PCR results [14], [15]. In order to ensure the robustness and reliability of the scientific methodology we established a PCR method where instead of selecting one XMRV target amplimer, three independent sets of primers were specifically selected to simultaneously amplify sequences from the XMRV *gag, pol* and *env* genes (Figure 1A). The three amplimers were 458, 510 and 782 base pairs respectively. Through optimization of hot-start and touch-down conditions, this triplex PCR method was able to detect all of these XMRV sequences in a single tube. Most importantly, this assay showed a high sensitivity for all three sequences with a detection limit as low as 5 pg genomic DNA per sample (Figure 1B). Partial amplifications of the *gag, pol* and *env* genes were also generated from as little as 0.05 pg of input DNA.

### Validation of DNA Sample Integrity

**Figure 2:**
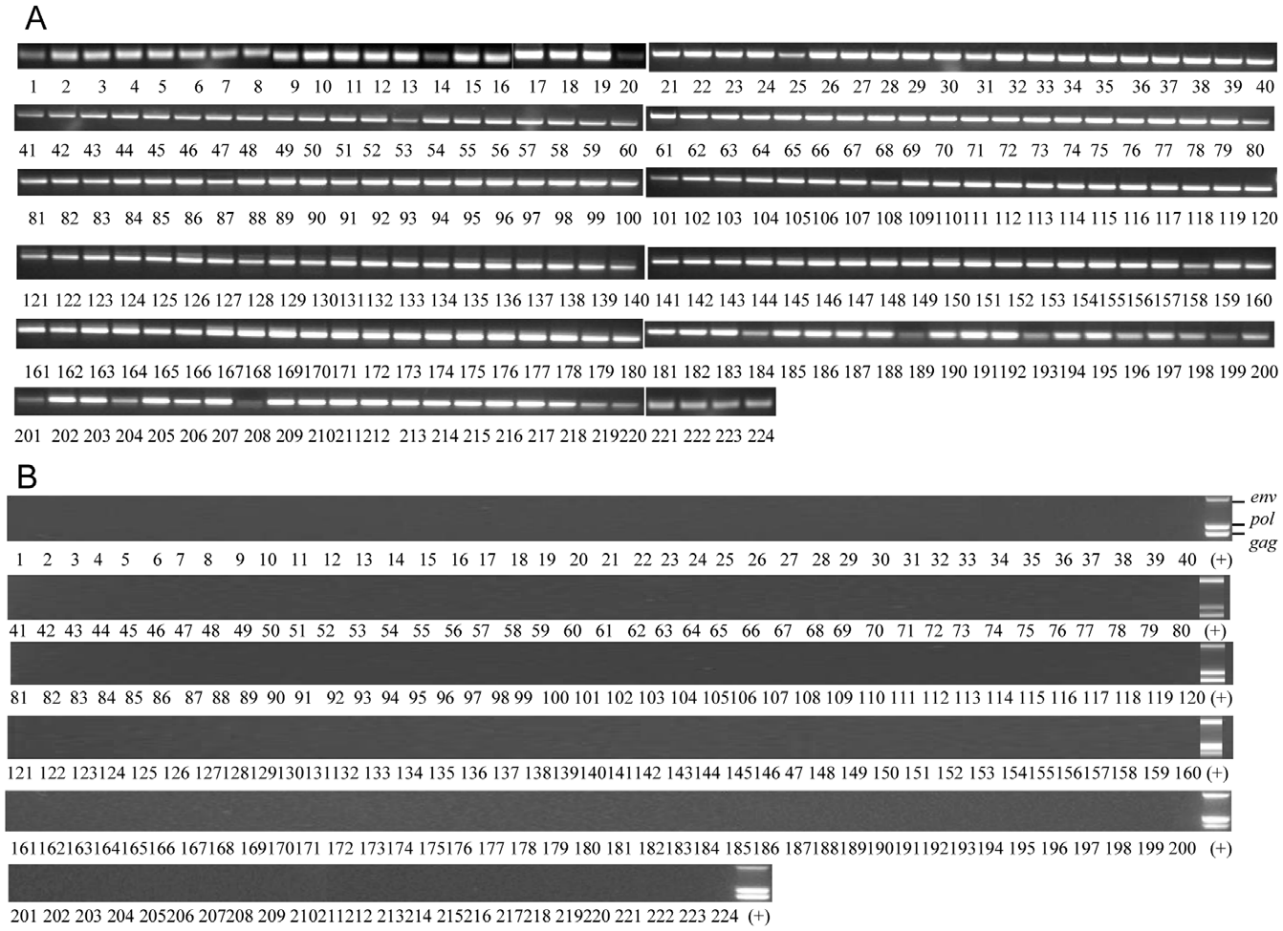
PCR analysis of XMRV and *GAPDH* in LBC samples. (A) PCR detection of the housekeeping gene *GAPDH* in 224 DNA preparations from liquid based cervical cytology using 33 cycles of amplification. (B) Triplex PCR detection of XMRV *gag, pol* and *env* gene sequences by hot-start touch-down, 45 cycles multiplex PCR using the prostate carcinoma cell line 22Rv1 DNA as a positive control.

The quality and integrity of all DNA preparations were verified by PCR amplification of the housekeeping gene *GAPDH,* which could effectively be amplified from all 224 LBC cervical smear sample DNA extracts as shown in Figure 2A.

### HIV/HPV Status of the Samples

The HIV status of all the patients was known from previous screening carried out at Kenyatta National Hospital. Cervical HPV status of LBC samples (including extensive HPV type analysis) was analysed in Manchester, UK (manuscript in preparation). Table 1 shows the sample set details in terms of HIV/HPV positivity and whether the HPV types present were considered high risk, low risk or both. For the purposes of this study high risk types encompassed types 16, 18, 31, 33, 35, 39, 45, 51, 52, 53, 56, 58, 59, 66, 68, 70, 73, 82 and low risk types 6, 11, 40, 42, 43, 44/55.

**Table 1:** HPV and HIV status of sample cohort.

	HIV (+) (n = 113)	HIV (−) (n = 110)
**HPV (+) HR**	50 (44.2%)	36 (32.7%)
**HPV (+) LR**	7 (6.3%)	9 (8.2%)
**HPV (+) HR/LR**	18 (15.9%)	1 (0.9%)
**HPV (−)**	38 (33.6%)	64 (58.2%)

Papillocheck analysis of HIV positive and negative women to determine the presence of high and low risk HPV infections. Of the 224 samples analysed one HIV negative sample failed quality control so is not included in the dataset. HPV types tested for were high risk 16, 18, 31, 33, 35, 39, 45, 51, 52, 53, 56, 58, 59, 66, 68, 70, 73, 82 and low risk types 6, 11, 40, 42, 43, 44/55 as defined by the Papillocheck system.

### Detection of XMRV by Triplex PCR in LBC samples

The cervical smear sample DNA was also tested for the presence of XMRV. As shown in Figure 2B XMRV *gag, pol* and *env* sequences were detected in the positive control DNA from the prostate cancer cell line 22Rv1 which has previously been shown to express multiple integrated copies of the virus [16]. XMRV sequences were not however detected in any of the 224 participant samples.

## Discussion

Depending on their mode of transmission retroviruses can be classified as exogenous or endogenous. Endogenous retroviruses and viral elements have been found in numerous vertebrate genomes and are generally non-pathogenic in their natural hosts. In humans and mice, for example, 8-10% of the genome is thought to be of viral origin. In some cases, however, endogenous retroviruses can cause disease. An example of this is the murine gammaretroviruses or murine leukaemia viruses, so called because of their association with haemopoietic malignancies. Gammaretroviruses usually transmit within a single host species [17], but there are a number of reported potential interspecies transmissions [18], [19]. One relatively recent example is that of koala retrovirus (KoRV), which causes lymphoid neoplasias and immunosupression in captive and wild koalas [20]. KoRv is closely related to XMRV which itself was thought to be a trans-species transmission of MLV and MLV-related viruses from mouse to humans.

There were several reasons why we felt it important to look for the presence of XMRV in cells of the cervix. Firstly no such study had yet been done despite the fact that XMRV could be transmitted via a sexual route in a similar fashion to the cervical cancer causing virus HPV or indeed, HIV. The case for examining XMRV in this sample set was therefore strong. In addition HIV-1 is known to facilitate infection of positive individuals with many opportunistic pathogens including HPV [11], [12]. XMRV was thus another potential candidate here. It was important that failure of many laboratories to find XMRV in other tissues should not influence or preclude examination of the cervix since the reproductive tract is known to be an area of anatomical sanctuary for viruses. Reservoirs of HIV for example are found in both male and female reproductive tract and indeed drug resistance is often a problem in this area [21], [22], [23]. Also despite endogenous retroviruses being quite ubiquitous in the genome it is known that many appear to concentrate expression in the reproductive tract [24], [25]. We consequently sought to provide a definitive answer as to the expression of XMRV in cervical cells and if so whether or not this could be related to HIV and/or HPV status.

We therefore designed three sets of primers specific for the XMRV *gag, pol,* and *env* genes and combined these with optimised hot-start and touch-down conditions. It was possible using this method to detect these three target genes in positive controls using a single run, simultaneous triplex PCR approach (Figure 1A). Our results with the sample set were very clear in that no evidence of XMRV was found in any of the samples tested. The HPV and HIV status did not influence the findings, indicating that neither virus increased susceptibility to XMRV.

Given the high sensitivity of the method employed (lower limit of detection for all three bands was routinely 5 pg of 22Rv1 DNA) we did not proceed to look for XMRV at the level of protein as this was deemed unnecessary. Similarly we did not carry out proposed RNASEL typing studies in support of the XMRV/RNASEL mutation link. Whilst recognising that it has been suggested that discrepancies of XMRV detection could be generated from viral sequence variation or geographic specificities [26], [27], the sensitivity and selectivity of the method used indicates that the complete absence of any detectable XMRV in our study is highly unlikely to be due to inadequate detection systems. Rather our results are consistent with the recent reports that XMRV is a laboratory generated recombinant virus [28], [29]. Furthermore the lack of XMRV in HIV infected individuals is supportive of several previous studies which failed to detect the gammaretrovirus in the peripheral blood mononuclear cells of this cohort [30], [31], [32].

To conclude, we did not find any evidence for XMRV in LBC cervical cells from 224 Kenyan women whether infected with HIV and/or HPV or not. It is not inconceivable that at some point in the future we may see a zoonotic transmission of MLV or indeed other mouse viruses into the human population. This may be of particular relevance to the population of women tested in this study since tropical Africa is one of several emerging disease hotspots [33], [34]. Given the apparent disposition of XMRV for cells of the simian reproductive tract perhaps we should periodically monitor such human tissues for emerging pathogens of this type.
